# Flavonoids from *Orostachys japonicus* A. Berger Inhibit the Invasion of LnCaP Prostate Carcinoma Cells by Inactivating Akt and Modulating Tight Junctions

**DOI:** 10.3390/ijms140918407

**Published:** 2013-09-06

**Authors:** Dong Yeok Shin, Won Sup Lee, Ji Hyun Jung, Su Hyun Hong, Cheol Park, Hye Jung Kim, Gi-Young Kim, Hye Jin Hwang, Gon Sup Kim, Jin-Myung Jung, Chung Ho Ryu, Sung Chul Shin, Soon Chan Hong, Yung Hyun Choi

**Affiliations:** 1 Dongnam Institute of Radiological & Medicine Sciences, Busan 619-953, Korea; E-Mail: bboglyang@hanmail.net; 2 Departments of Internal Medicine, Institute of Health Sciences, Gyeongsang National University School of Medicine, Jinju 660-702, Korea; E-Mail: henia1122@hanmail.net; 3 Department of Biochemistry, Dongeui University College of Oriental Medicine and Anti-Aging Research Center & Blue-Bio Industry RIC, Dongeui University, Busan 614-052, Korea; E-Mail: hongsh@deu.ac.kr; 4 Department of Molecular Biology, Dongeui University, Busan 614-714, Korea; E-Mail: leoleo1969@hotmail.com; 5 Departments of Pharmacology, Institute of Health Sciences, Gyeongsang National University School of Medicine, Jinju 660-702, Korea; E-Mail: curlysookim@hanmail.net; 6 Laboratory of Immunobiology, Department of Marine Life Sciences, Jeju National University, Jeju 690-756, Korea; E-Mail: immunkim@jejunu.ac.kr; 7 Department of Food and Nutrition, Dongeui University, Busan 614-714, Korea; E-Mail: hhj2001@deu.ac.kr; 8 School of Veterinary Medicine, Research Institute of Life Science, Gyeongsang National University, Jinju 660-701, Korea; E-Mail: gonskim@gnu.ac.kr; 9 Departments of Neurosurgery, Institute of Health Sciences, Gyeongsang National University School of Medicine, Jinju 660-702, Korea; E-Mail: gnuhjjm@nongae.gsnu.ac.kr; 10 Division of Applied Life Science (BK 21 Program), Institute of Agriculture and Life Science, Gyeongsang National University, Jinju 660-701, Korea; E-Mail: ryu@gsnu.ac.kr; 11 Department of Chemistry, Research Institute of Life Science, Gyeongsang National University, Jinju 660-701, Korea; E-Mail: scshin@gsnu.ac.kr; 12 Departments of Surgery, Institute of Health Sciences, Gyeongsang National University School of Medicine, Jinju 660-702, Korea; E-Mail: hongsc@nongae.gsnu.ac.kr

**Keywords:** *Orostachys japonicus*, flavonoids, LnCaP cells, tight junctions, Akt

## Abstract

Tight junctions (TJs) are a mode of cell-to-cell adhesion in epithelial or endothelial cells, and serve as a physical barrier to maintenance of homeostasis in body by controlling paracellular transport. Claudins are the most important molecules of the TJs, but paradoxically these proteins are frequently over-expressed in cancers and their overexpression is implicated in the invasive potential of cancer. Hence, we investigated the effects of flavonoids extracted from *Orostachys japonicus* A. Berger (FEOJ) on TJs and the expression of claudins as well as cancer invasion along with in LnCaP human prostate cancer. FEOJ suppressed cancer cell motility and invasiveness at the concentrations where FEOJ did not show anti-proliferative activity. FEOJ increased transepithelial electrical resistance (TER) associated with tightening TJs, and suppressed expression of claudin proteins. Furthermore, FEOJ suppressed the activities of MMP-2 and -9 in a dose-dependent manner, which came from the activation of tissue inhibitor of metalloproteinases (TIMPs) by FEOJ. FEOJ suppressed migration and invasion by suppressing PI3K/Akt signaling pathway. Taken together, this study suggest that FEOJ suppresses cancer migration and invasion by tightening TJs through the suppression of claudin expression, and by suppressing MMPs in LnCaP human prostate cancer cells, which at least in part results from the suppression of PI3K/Akt signaling pathway.

## 1. Introduction

Tight junctions (TJs) represent one mode of cell-to-cell adhesion in epithelial or endothelial cells, establishing contact between adjacent cells. They serve as a physical barrier to prevent solutes and water from passing freely through the paracellular space contributing to maintenance of epithelial cell polarity [1]. TJs become disorganized or lost while normal epithelial cells transforming into precancerous or cancerous cells [2], and the alteration and loss of cell polarity are hall marks for tumorigenesis of epithelial cell. Altered TJs lead to decrease in resistance to electrical current (transepithelial electrical resistance; TER) and increase in paracellular permeability [2].

Claudins are 20–27 kDa small transmembrane proteins serving as key integral membrane proteins forming the backbone of TJs [3,4]. They form homodimers or heterodimers to produce paired strands between neighboring cells, establishing the barrier and controlling the transport of molecules in the intercellular space between cells. They have four transmembrane domains, with the *N*-terminus and the C-terminus in the cytoplasm [5]. However, evidence suggested that these proteins be frequently over-expressed in cancers [6–8] and associated with invasive potential of cancer cells [9] even though how the over-expressed claudins enhance the invasive potential is not fully elucidated.

In addition, the over-expressed claudin expression in cancer is associated with increased MMP’s activity [9]. Matrix metalloproteinases (MMPs) also play a crucial role in cancer invasion, by interacting with a broad spectrum of cell surface molecules and destroying extracellular matrix (ECM) substrates. Particularly, MMP-2 (gelatinase-A) and MMP-9 (gelatinase-B) are highly expressed in epithelial cancer cells [10] and involved in epithelial-mesenchymal transition (EMT) implicated in cancer invasion and metastasis [11,12].

Recently, the dietary agents and some phytochemicals are known to safely modulate physiological function and enhance anti-cancer activity with minimal toxicity, which have drawn our interests to seek the possibility of opening a new paradigm for cancer prevention or treatment [13]. *Orostachys japonicus* A. Berger, a member of the family Liliaceae, has been used as a folk remedy for cancer treatment. The extract of *Orostachys japonicus* A. Berger was used as an adjuvant or an alternative treatment for the chemotherapy. The extract can be easily obtained by on-line in Korea. However, few data are available on the anti-cancer effects of the extract of *Orostachys japonicus* A. Berger [14]. Therefore, we investigated the effects of flavonoids extracted from *Orostachys japonicus* A. Berger (FEOJ) on invasive parameters and tightness of TJs along with TJ-associated proteins, using human prostate carcinoma cell lines.

## 2. Results and Discussion

### 2.1. FEOJ Tightened TJs by Suppressing the Expressions of Claudin-1 and Claudin-3 at the Transcriptional Level in LnCaP Human Prostate Cancer Cells

**Figure 1:**
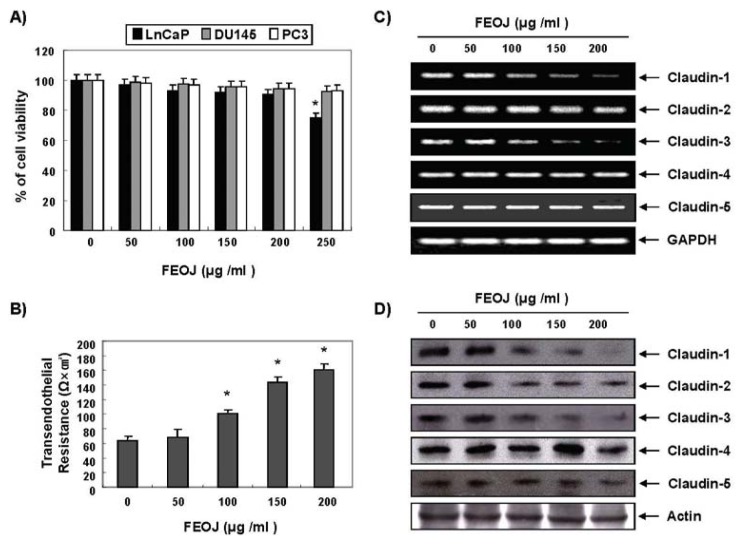
Tightening tight junctions (TJs) by flavonoids extracted from *Orostachys japonicus* (FEOJ) through suppression of the expressions of claudin-1 and claudin-3 at the transcriptional level in LnCaP human prostate cancer cells. Du145, LnCaP, and PC3 human prostate carcinoma cells were treated with FEOJ at the indicated concentrations for 48 h. (**A**) Cell viability was estimated by MTT assay; (**B**) TER values were measured. Each point represents the mean ± SD of three independent experiments. Significance was determined by the Student’s *t*-test (*p < 0.05 *vs.* control); (**C**) Total RNA was isolated and reverse-transcribed. Resulting cDNAs were then subjected to PCR. The reaction products were subjected to electrophoresis in a 1% agarose gel and visualized by EtBr staining. GAPDH was used as an internal control; and (**D**) Western blotting was performed using the indicated antibodies and an ECL detection system. Actin was used as an internal control. Data were representative of two independent experiments.

Prostate cancer is one of the most common types of cancer in men in the world, and the incidence of prostate cancer is increasing in Korea [15,16]. Firstly, we investigate the effects of FEOJ on cell viability with three human prostate cancer cells (Du145, LnCaP, and PC3 cells). MTT assays revealed that LnCaP cells were most sensitive to FEOJ at various concentrations of FEOJ while any significant inhibitory effects were not observed up to the concentration of 250 μg/mL in the other two cell lines (Figure 1A). We chose LnCaP cells for the further treatment.

To determine whether FEOJ tighten TJs, we measured transephithelial electrical resistance (TER), which is a measure for tightness of tight junction. As shown in Figure 1B, FEOJ significantly increased their TER levels in a dose-dependent manner, suggesting that FEOJ tightens TJs in LnCaP cells (approximately 1.6-fold and 2.4-fold by 100 and 200 μg/mL of FEOJ, respectively). Next, we investigated the key genes that involved in tight junction formation, claudins. RT-PCR analyses revealed that the mRNA levels of claudin-1, claudin-2 and claudin-3 were suppressed by FEOJ (Figure 1C). Western blot analyses confirmed that the above findings (Figure 1D). These findings suggested that FEOJ tightened TJs by suppressing the mRNA levels of claudin-1, claudin-2 and claudin-3 in LnCaP human prostate cancer cells.

### 2.2. FEOJ Inhibited Expression of MMPs and Induced TIMPs at the Transcriptional Level in LnCaP Cells, Which Might Be Associated with the Anti-Invasive Effects of FEOJ

**Figure 2:**
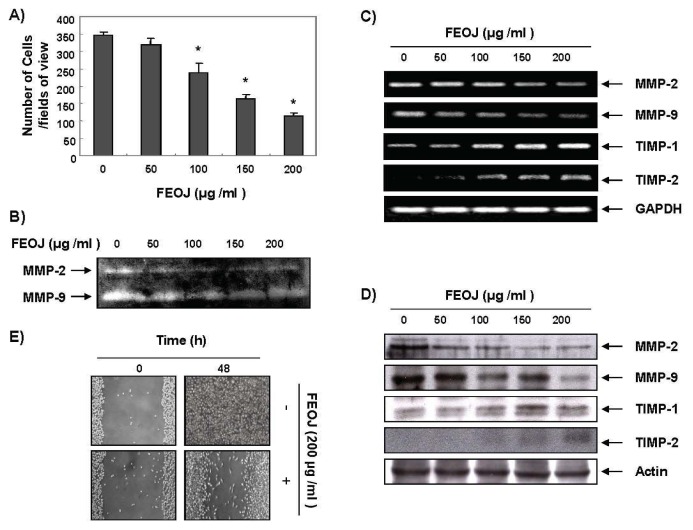
The inhibitory effects of FEOJ on invasion of LnCaP cells by inhibiting expression of MMPs and inducing TIMPs both at the transcriptional level. (**A**) Effects on invasion of LnCaP cells. The cells (5 × 10^4^ cells) were loaded on pre-coated Matrigel 24-well invasion chambers (BD Biosciences) in serum-free medium containing FEOJ. The cells were treated with FEOJ for 48 h in a Matrigel-coated transwell. Data were expressed as the mean of triplicate samples. Significance was determined by the Student’s *t*-test (*p < 0.05 *vs.* control); (**B**) MMP-2 and MMP-9 protein levels were measured by gelatin zymography. Cells were incubated at the indicated concentrations for 48 h; (**C**) Total RNA was isolated and reverse-transcribed. Resulting cDNAs were then subjected to PCR. The reaction products were subjected to electrophoresis in a 1% agarose gel and visualized by EtBr staining. GAPDH was used as an internal control; (**D**) Western blotting was performed using the indicated antibodies and an ECL detection system. Actin was used as an internal control; and (**E**) Cells were grown to 100% confluent monolayer on 30-mm cell culture dishes; a scratch was then made through the cell layer using a pipette tip. After washing with PBS, serum-free media (to prevent cell proliferation) containing either vehicle or FEOJ (200 μg/mL) was added for 48 h. Photographs were taken for evaluation of cell movement into the wounded area. Data were representative of two independent experiments.

Evidence suggested that the inhibition of claudins can reduce cancer invasive potential in cancer cells, and that over-expressed claudin expression in cancer is associated with increased MMP activity [9]. Hence, we next investigated whether FEOJ inhibits cancer cell invasion. As shown in Figure 2A, FEOJ significantly inhibited LnCaP cell invasion in a dose-dependent manner as measured by Matrigel invasion assays. The cancer invasion is primarily mediated by the gelatinase MMPs. We assessed the effects of FEOJ on the expression of MMPs by gelatin zymography and Western blot analyses. Gelatin zymography revealed that the activities of MMP-2 and -9 in LnCaP cells were significantly decreased by FEOJ treatment in a dose-dependent manner (Figure 2B), which was connected with the down-regulation of their mRNA and protein levels (Figure 2C,D). Their endogenous inhibitors, tissue inhibitors of metalloproteinase (TIMPs), play critical roles during epithelial-meshnchymal transition (EMT) that is involved in EMT [17]. We also investigated expression of TIMPs. Unexpectedly, we found that FEOJ significantly increased the TIMP-1 and TIMP-2 mRNA and protein in a dose-dependent manner. These results suggested that the anti-invasive effect of FEOJ was associated with down-regulation of *MMP-2* and *MMP-9* expressions and up-regulation of *TIMP-1* and *TIMP-2* expression both at transcriptional level.

Next we performed wound healing test to check the effects of FEOJ on cancer cell migration, because the migration of cells is also an important process for the invasion of cancer cells. The results demonstrated that 200 μg/mL of FEOJ, where no cytotoxicity was shown by MTT assay, delayed the cell motility of LnCaP cells compared to that of control cells (Figure 2E). These findings suggested that FEOJ inhibited cell invasion by suppressing MMPs through the induction of TIMPs at the transcriptional level in LnCaP cells.

### 2.3. FEOJ Inhibits Migration and Invasion and at Least in Part by Suppressing Phosphatidylinositide 3-Kinase (PI3K)/Akt Pathway

**Figure 3:**
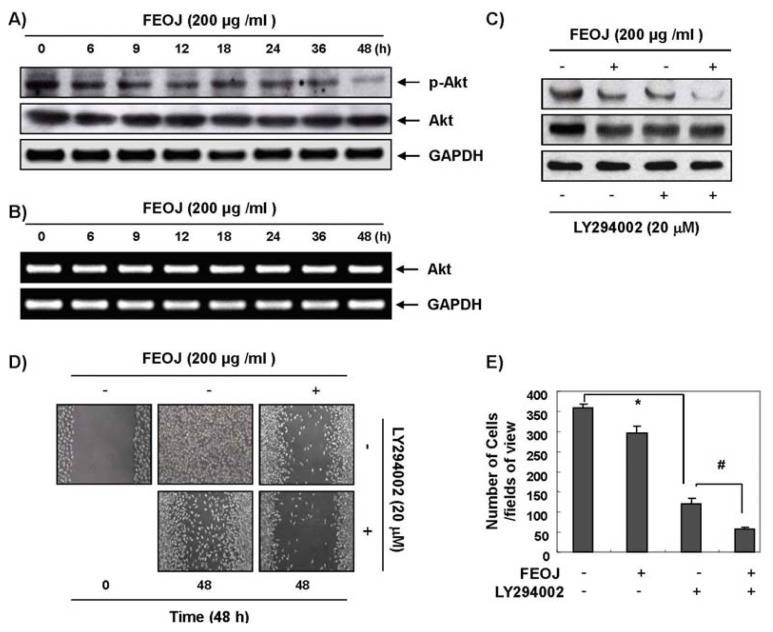
The inhibitory effects of FEOJ on cancer cell migration and invasion by suppression of PI3k/Akt pathway. LnCaP cells were treated with FEOJ and/or LY294002 at the indicated concentrations for 48 h. (**A**,**C**) Western blotting was performed using the indicated antibodies and an ECL detection system. Actin was used as an internal control; (**B**) Total RNA was isolated and reverse-transcribed. Resulting cDNAs were then subjected to PCR. The reaction products were subjected to electrophoresis in a 1% agarose gel and visualized by EtBr staining. GAPDH was used as an internal control; (**D**) Cells were grown to 100% confluent monolayer on 30-mm cell culture dishes; a scratch was then made through the cell layer using a pipette tip. After washing with PBS, serum-free media (to prevent cell proliferation) containing vehicle or FEOJ and/or LY294002 was added for 48 h. Data were representative of two independent experiments; and (**E**) Effects on invasion of LnCaP cells. The cells (5 × 10^4^ cells) were loaded on pre-coated Matrigel 24-well invasion chambers (BD Biosciences, San Jose, CA, USA) in the presence or absence of FEOJ and/or LY294002. The cells were treated with FEOJ for 48 h in a Matrigel-coated transwell. Data were expressed as the mean of triplicate samples. Significance was determined by the Student’s *t*-test (*p < 0.05 *vs.* control, and ^#^*p* < 0.05 *vs.* FEOJ treatment).

PI3k/Akt pathway is activated in cancer cells, and is involved cell proliferation, motility, and survival by regulating the expression of proteins involved in EMT and metastasis. To investigate the possible mechanism of FEOJ-induced anti-invasion and migration, we assessed the changes in phosphoryaltion of Akt after FEOJ treatment. The phosphoryatlion of Akt was inhibited by FEOJ in a dose-dependent manner (Figure 3A). The total level of Akt was also slightly changed after FEOJ treatment, but the level of Akt at mRNA remained unchanged (Figure 3B). To confirm that the association between the inhibitory effects of FEOJ on PI3k/Akt pathway and the anti-invasive effects of FEOJ, we performed Matrigel invasion assays and wound healing test with LY294002, a PI3k/Akt inhibitor. As shown in Figure 3C, a concentration of 20 μM of LY294002 was adequate to test additive inhibitory effects on PI3k/Akt pathway. Both the wound healing test (Figure 3D) and invasion test (Figure 3E) revealed that FEOJ in combination of LY294002 further inhibited the migration and invasion of cancer cells than did FEOJ alone. These findings suggested that compare to with and at least in part by suppressing PI3k/Akt pathway.

### 2.4. Discussion

This study was designed to determine the anti-cancer effects of FEOJ on tight junctions (TJs) and the expression of claudins as well as the expression of MMPs. We found that FEOJ have anti-invasive activities not only through modulation of tightening TJs by suppressing the expression of claudins, but also through inhibition of MMP-2 and MMP-9 expression in LnCaP human prostate cancer cells. Even though claudins serves as a major component TJ protein, paradoxically the claudins are over-exprressed in cancer and their over-expressed claudins may result in dysfunctional TJs [6–8]. The previous study showed that TJs might be tightened by the suppression of the claudins in cancer [18]. In addition, the loosened TJs probably due to the alteration and loss of cell polarity were found in cancer tissue compared to normal tissue. Actually, TER values can be used as a measure for the degree of tightness of TJ in other studies [19], and were significantly lower in cancer tissue than in normal tissue. Therefore, the increase in TER by FEOJ indicated the increase in tightness of TJs. The increased tightness may contribute to anti-invasive activity of FEOJ, because the disruption of TJs with deregulation of TJ proteins is an early event of cancer invasion and metastasis and the inhibition of claudin-3 and -4 reduced cancer invasive potential in cancer cells [9]. In addition, claudin-1, -3 and -4 are frequently over-expressed in cancer cells [6–8], and the over-expression of claudin-3 and -4 is associated with increased MMP-2 activity [9]. These findings also support that the suppression of claudins and MMPs by FEOJ can be associate with the anti-invasive activity of FEOJ.

Here, we also demonstrated that FEOJ inhibit invasion and migration by inhibiting PI3/Akt pathway. The phosphorylation of Akt was suppressed by FEOJ as early as 6 h, but it takes 48 h to completely suppress Akt activity (Figure 3A). This finding suggests that have the chance of having other mechanisms for the effects of FEOJ on TER or claudin expression. However, in the previous study we demonstrated that the inhibition of PI3/Akt pathway could lead to the suppression of claudin-1 and claudin-3 expression [18]. In that context, we may conclude that FEOJ suppresses cancer migration and invasion by tightening TJs through the suppression of claudin and MMP expression at least in part by suppressing PI3K/Akt signaling pathway. However, we here did not clearly demonstrate whether FEOJ inhibited MMP-2 and MMP-9 activity by inhibiting the expression of claudins. We need further investigation on this point.

The inhibitory effects of FEOJ on *MMP-2* and *MMP-9* have another significant meaning. For cancer invasion, proteolytic digestion of the extracellular matrix (ECM) and cell migration are essential processes. In proteolytic digestion of ECM and cell migration, *MMP-2* and *MMP-9* plays an important role [20,21]. For these reasons, a lot of effort has been devoted to develop MMP inhibitors because cancer invasion is prerequisite for cancer metastasis that is the ultimate cause of cancer death. Here, we found that FEOJ down-regulate *MMP-2* and *MMP-9* expressions, and up-regulated *TIMP-1* and *TIMP-2* genes that are specific inhibitors that bind MMPs in a 1:1 stoichiometry.

However, the finding that FEOJ down-regulate *MMP-2* and *MMP-9* genes, and up-regulated *TIMP-1* and *TIMP-2* genes is not easily explicable because of the following reasons. The effect of *TIMP-2* on *MMP2* activity varies depending on the expression levels of *TIMP-2* [22], and further pro-MMP-2 is activated by the assistance of low concentration of *TIMP-2* [23]. In addition, the expression *TIMP-1* and *TIMP-2* in cancer as well as the relationships between *MMPs* and *TIMPs* may vary depending on cell types [24]. Furthermore, co-incidental increase in *TIMPs* and *MMPs* expression was frequently found in cancer tissue [25,26].

Based on modern knowledge, the up-regulated *TIMP-1* and *TIMP-2* expression by FEOJ inhibits the enzymatic activity of *MMP-2* and *MMP-9* can be understandable. However, FEOJ also inhibited *MMP-2* and *MMP-9* expression at the transcriptional level. That means the down-regulation of *MMP-2* and *MMP-9* expression did not resulted from FEOJ-induced up-regulation of *TIMP-1* and *TIMP-2* expression itself even though there is a report that the agent that have an inhibitory effect on PI3K/Akt pathway show the exactly same results [27]. Therefore, we need to further detailed experiment by silencing each gene to elucidate the mechanisms. Although not all these results are fully understood at present, it is evident that the suppression of PI3K/Akt pathway is an important upstream signaling pathway for the inhibitory effects of FEOJ on cancer invasion and migration, and that FEOJ suppressed claudin-1 and claudin-3 and tightened TJs. The concentrations used in the present study are consistent with those in another study of the anti-cancer effect of FEOJ in wild-type P53-haboring AGS cells [14]. In the study, FEOJ showed cytotoxicity in AGS cells whereas FEOJ did not show a significant cytotoxicity. Considering the extract of *Orostachys japonicus* A. Berger used in human as folk medicine usually tolerable, the concentration should be ideal when the significant cytotoxicity of the FEOJ is not observed although the anticancer effects is shown like this study.

Final limitation of our study is that we tested the effects of FEOJ that contains many phytochemicals, but that we do not know exactly which components are essential components for these activities because few results on the effect of FEOJ are available for cancer cells [14]. We only know that FEOJ has these actions. We are now investigating whether the *Orostachys japonicus* A. Berger, has anti-cancer effects. We are just providing evidence that *Orostachys japonicus* A. Berger, has anti-invasive effects on human prostate cancer cells. The further investigation is also required to suggest which molecule is showing these effects.

## 3. Experimental Section

### 3.1. Cell Culture

Du145, LnCaP, and PC3 human prostate carcinoma cells were obtained from American Type Culture Collection (Rockville, MD, USA) and grown in RPMI 1640 supplemented with 10% fetal bovine serum (FBS), 2 mM L-glutamine, 25 mM *N*-2-hydroxyethylpiperazine-*N′*-2-ethanesulfonic acid, 25 mM NaHCO_3_, 100 IU/mL penicillin and 10 μg/ml streptomycin at 37 °C in a humidified atmosphere of 95% air and 5% CO_2_. FEOJ were a generous gift from Dr. S.C. Shin (Department of Chemistry, Gyeongsang National University, Jinju, Korea). FEOJ contains following flavonoids; (1): Procyanidin; (2): Epigallocatechin; (3): Epicatechin; (4): Quercetin; (5): Kaempferol; (6): Myricetin; (7): Kaempferol; and (8): coumaroylspermidine. The major component was Quercetin.

### 3.2. MTT Assay

For the cell viability assay, the cells were plated at 3 × 10^5^ cells/well in RPMI 1640 medium containing FEOJ in a 6-well microtiter plate (NUNC™, Roskilde, Denmark). After incubation with various concentrations of FEOJ for 48 h, cell viability was determined using the 3-(4,5-dimethylthiazol-2-yl)-2,5-diphenyltetrazolium bromide (MTT) assay, which is based on the conversion of MTT to MTT-formazan by mitochondria.

### 3.3. Wound Healing Assay

LnCaP cells were plated at 5 × 10^5^ cells/well and grown to 100% confluent monolayer on 30-mm cell culture dishes coated with rat tail collagen (20 μg/mL, BD Biosciences, Bedford, MA, USA). Confluent cells were wounded by scraping with a pipette tip. After the cells were rinsed three times with culture medium, the cells were incubated with 1% FBS-containing medium supplemented with the indicated concentration of FEOJ for 48 h. The images were recorded at 48 h after scratch using an Olympus photomicroscope at 40× magnification.

### 3.4. Cell Invasion Assay

The cells were cultured in serum-free media overnight. Cells (5 × 10^4^ cells) were loaded onto pre-coated Matrigel 24-well invasion chambers (BD Biosciences, San Jose, CA, USA) in the presence or absence of FEOJ (200 μg/mL). Then 0.5 mL of 5% fetal calf serum medium was added to the wells of the plate to serve as the chemoattractant. The Matrigel invasion chambers were incubated for 24 h. Invading cells were fixed with 10% formalin, stained with a hematoxylin and Eosin Y and counted.

### 3.5. Gelatin Zymography

The gelatinolytic activities for MMP-2 and MMP-9 in the culture medium were assayed by electrophoresis on 8% polyacrylamide gels containing 1 mg/mL gelatin at 4 °C Polyacrylamide gels were run at 120 V, washed in 2.5% Triton X-100 for 1 h, and then incubated for 24 h at 37 °C in activation buffer (50 mM Tris–HCl, pH 7.5, 10 mM CaCl_2_). Then, the gels were stained with 0.5% (*w*/*v*) Coomassie brilliant blue G-250 for 1h, then lightly destained in methanol:acetic acid:water (3:1:6). White lysis zones indicating gelatin degradation were revealed by staining with Coomassie brilliant blue [28].

### 3.6. Measurement of Transepithelial Electrical Resistance (TER)

TER was measured with an EVOM Epithelial Tissue Voltohmmeter (World Precision Instruments, Sarasota, FL, USA), equipped with a pair of STX-2 chopstick electrodes. Briefly, LnCaP cells were seeded into the insert of a Transwell^®^ with 8.0 μm pore (upper chamber) (Corning Costar Corp., Corning, NY, USA) and allowed to reach full confluence. Then, the fresh medium was replaced for further experiments. The cells were treated with FEOJ for 48 h. Electrodes were placed at the upper and lower chambers, and resistance was measured with the voltohmmeter.

### 3.7. RNA Extraction and Reverse Transcription-PCR (RT-PCR)

Total RNA was isolated from the LnCaP cells (treated and untreated) using an RNeasy kit (Qiagen, La Jolla, CA, USA). An aliquot of 0.5 μg of RNA was used for reverse transcription cDNA synthesized from 2 μg of total RNA using Avian Myeloblastosis Virus (AMV) Reverse Transcriptase (Amersham Corp., Arlington Heights, IL, USA). The mRNAs were amplified by PCR with the indicated primer sets (Table 1). The conditions for PCR reaction were 1 × (94 °C, 3 min); 35 × (94 °C, 45 s; 58 °C, 45 s; and 72 °C, 1 min); and 1 × (72 °C, 10 min). Amplification products obtained by PCR were electrophoretically separated on 1% agarose gel and visualized by ethidium bromide (EtBr) staining.

### 3.8. Western Blot Analysis

Total cell lysates were prepared in lysis buffer containing 0.5% SDS, 1% NP-40, 1% sodium deoxycholate, 150 mM NaCl, 50 mM Tris-HCl (pH 7.5), and protease inhibitors. The concentrations of cell lysate proteins were determined by the Bradford protein assay (Biorad lab, Richmond, CA, USA) using bovine serum albumin as the standard. Molecular mass markers for proteins were purchased from Pharmacia Biotech (Saclay, France). For Western blotting, 30 micrograms of proteins were resolved by electrophoresis, electrotransferred to polyvinylidene difluoride membranes (Millipore, Bedford, MA, USA), and then incubated with the indicated primary antibodies followed by secondary antibody conjugated to peroxidase. Blots were developed with an ECL detection system.

### 3.9. Statistical Analysis

All data represent means ± standard deviations. Statistical significance was determined using the one-way analysis of variance (ANOVA) with post-test Neuman-Keuls for more than two groups and Student’s *t*-test for two groups. *p* < 0.05 was accepted as statistically significant.

## 4. Conclusions

**Figure 4:**
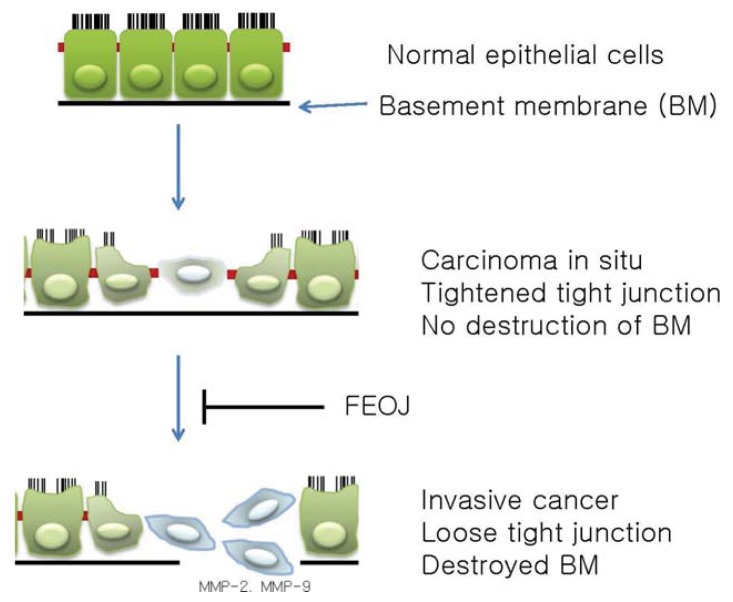
Schematic representation on the anti-cancer effects of FEOJ in human prostate cancer cells. Tight junctions (TJs) and polarity of the normal cells become disorganized or lost during carcinogenesis from normal epithelial cells to cancer cells. To metastasize to farther regional nodes and distant organs, cells need to destroyed basement membrane (BM) and extracellular matrix (ECM). Matrix matalloproteinases (MMPs) play a critical role in ECM and BM degradation. Among them, MMP-2 and MMP-9 are important in metastasis. FEOJ suppresses cancer migration and invasion by suppressing MMPs and tightening TJs in LnCaP human prostate cancer cells.

In this study, we demonstrated that FEOJ tightened TJs and have anti-invasive properties in LnCaP human prostate cancer cells. These activities are mediated at least in part by suppression of claudin-1 and claudin-3 as well as by suppression of *MMP-2* and *MMP-9* expressions which are probably mediated through the inhibition of PI3K/AKT pathway. This study provides evidence that FEOJ might be useful phytochemicals for inhibiting invasion and migration of prostate cancer (Figure 4). However, additional further studies are needed to establish the mechanisms for the inhibitory effects on cancer invasion by up-regulation of *TIMP-1* and *TIMP-2*.

## Figures and Tables

**Table 1:** Sequence of primers used for RT-PCR.

Gene name	Orientation	Sequence
*MMP-2*	Sense	5′-CTT-CTT-CAA-GGA-CCG-GTT-CAT-3′
	Antisense	5′-GCT-GGC-TGA-GTA-GAT-CCA-GTA-3′

*MMP-9*	Sense	5′-TGG-GCT-ACG-TGA-CCT-ATG-ACC-AT-3′
	Antisense	5′-GCC-CAG-CCC-ACC-TCC-ACT-CCT-C-3′

*TIMP-1*	Sense	5′-TGG-GGA-CAC-CAG-AAG-TCA-AC-3′
	Antisense	5′-TTT-TCA-GAG-CCT-TGG-AGG-AG-3′

*TIMP-2*	Sense	5′-GTC-AGT-GAG-AAG-GAA-GTG-GAC-TCT-3′
	Antisense	5′-ATG-TTC-TTC-TCT-GTG-ACC-CAG-TC-3′

*Claudin-1*	Sense	5′-TCA-GCA-CTG-CCC-TGC-CCC-AGT-3′
	Antisense	5′-TGG-TGT-TGG-GTA-AGA-GGT-TGT-3′

*Claudin-2*	Sense	5′-ACA-CAC-AGC-ACA-GGC-ATC-AC-3′
	Antisense	5′-TCT-CCA-ATC-TCA-AAT-TTC-ATG-C-3′

*Claudin-3*	Sense	5′-AAG-GCC-AAG-ATC-ACC-ATC-GTG-3′
	Antisense	5′-AGA-CGT-AGT-CCT-TGC-GGT-CGT-3′

*Claudin-4*	Sense	5′-TGG-ATG-AAC-TGC-GTG-GTG-CAG-3′
	Antisense	5′-GAG-GCG-GCC-CAG-CCG-ACG-TA-3′

*Claudin-5*	Sense	5′-ACT-GCC-TTC-CTG-GAC-CAC-AA-3′
	Antisense	5′-CCC-CTT-CCA-AGT-CGT-CTG-C-3′

*Akt*	Sense	5′-GAA-CCG-TGT-CCT-GCA-GAA-CTC-TAG-3′
	Antisense	5′-GTG-GGT-CTG-GAA-TGA-GTA-CTT-GAG-3′

*GAPDH*	Sense	5′-CGG-AGT-CAA-CGG-ATT-TGG-TCG-TAT-3′
	Antisense	5′-AGC-CTT-CTC-CAT-GGT-GGT-GAA-GAC-3′
